# Autism Spectrum Disorder: Focus on Glutamatergic Neurotransmission

**DOI:** 10.3390/ijms23073861

**Published:** 2022-03-31

**Authors:** Martina Montanari, Giuseppina Martella, Paola Bonsi, Maria Meringolo

**Affiliations:** 1 Laboratory of Neurophysiology and Plasticity, IRCCS Fondazione Santa Lucia, 00179 Rome, Italy; martina.montanari@students.uniroma2.eu (M.M.); g.martella@hsantalucia.it (G.M.); 2 Department of Systems Neuroscience, University Tor Vergata, 00133 Rome, Italy

**Keywords:** brain development, ASD genes, animal model, signaling, therapy, glutamate receptors, metabolism, gut

## Abstract

Disturbances in the glutamatergic system have been increasingly documented in several neuropsychiatric disorders, including autism spectrum disorder (ASD). Glutamate-centered theories of ASD are based on evidence from patient samples and postmortem studies, as well as from studies documenting abnormalities in glutamatergic gene expression and metabolic pathways, including changes in the gut microbiota glutamate metabolism in patients with ASD. In addition, preclinical studies on animal models have demonstrated glutamatergic neurotransmission deficits and altered expression of glutamate synaptic proteins. At present, there are no approved glutamatergic drugs for ASD, but several ongoing clinical trials are currently focusing on evaluating in autistic patients glutamatergic pharmaceuticals already approved for other conditions. In this review, we provide an overview of the literature concerning the role of glutamatergic neurotransmission in the pathophysiology of ASD and as a potential target for novel treatments.

## 1. Introduction

Autism Spectrum Disorder (ASD) is a mosaic of neurodevelopmental conditions, which show common deficits in two behavioral domains: (i) social interaction and communication difficulties, (ii) narrow interests, and repetitive and stereotyped behaviors (DSM-5) [1,2]. ASD can be associated with several co-occurring conditions, including seizures, attention-deficit/hyperactivity disorder (ADHD), and other cognitive impairments [3,4]. The estimated prevalence of ASD is about 1% of the human population [5], with males affected four times more frequently than females [6].

Although it has become clear that ASD has a complex and multifactorial etiopathogenesis, twin and family studies indicate a strong genetic background and high heritability, with a concordance rate of 60% to 95% between monozygotic twins versus 0% to 30% between dizygotic twins [7]. Despite the high heterogeneity between ASD cases, the occurrence of shared symptoms suggests common deficits in some neurodevelopmental pathways. Neurotransmitters play a critical role in developing both the peripheral and the central nervous systems. It is therefore conceivable that neurotransmitter dysfunctions may be involved in ASD pathophysiology. Among neurotransmitters, glutamate (Glu) is considered a good candidate [8,9,10] as it is directly involved in brain development and synaptogenesis [11,12], memory, behavior, and motor activity regulation [13,14,15], and gastrointestinal functions [16,17]. Studies on postmortem or body fluids samples provide important evidence about critical changes in Glu concentration in both pediatric and adult patients with ASD [18,19]. Furthermore, abnormalities in Glu receptors genes and deregulation of glutamatergic pathways have been reported both in ASD patients and animal models [20,21,22,23]. In general, excitotoxicity has already been connected with ASD. In addition, the increased prevalence of epilepsy in autistic patients compared to the general population [24,25,26] further strengthens the hypothesis of the dysfunction in excitatory and/or inhibitory network activity. In the past decades, two opposite theories about the role of Glu signaling in ASD have been proposed. In 1998, Carlsson suggested that ASD might result from a decrease in Glu signaling according to (i) overlapping between the ASD condition and the symptoms produced by Glu antagonist, (ii) the neuroanatomical and neuroimaging evidence of glutamatergic areas impairment in ASD patients, (iii) the similarities with ASD symptoms in hypoglutamatergic animals treated with NMDA antagonists [27]. Conversely, Fatemi proposed an opposite theory, suggesting an hyperglutamatergic state in ASD, based on the observation of increased Glu levels in blood samples [19].

In this review, starting from the signaling of Glu in typical brain development, we will present current evidence supporting Glu involvement in ASD pathophysiology, including reports on alterations in intestinal Glu metabolism and ongoing clinical trials for the treatment of the core symptoms of the spectrum.

## 2. The Critical Role of the Glutamatergic System in Brain Development

**Figure 1:**
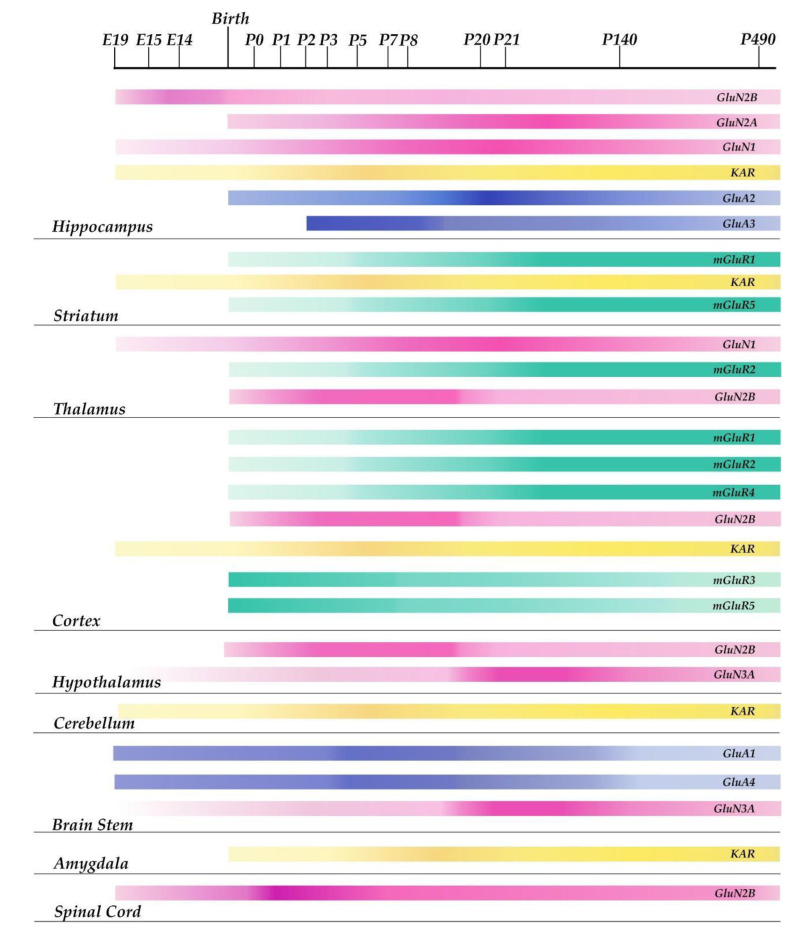
Schematic representation of the developmental time course of glutamatergic receptor subunits expression in different rodent brain areas. The variations in color intensity within the single bars represent changes along time in the expression levels of each receptor subunit in a particular area of the rodent brain. The midbrain, pons, and medulla are represented collectively as brain stem in the figure.

ASD symptoms appear early in development, within the first three years of life, during the fundamental period of rapid synapse formation and maturation [28,29]. Brain development involves a number of processes, including synaptogenesis, axonal and dendritic arborization, migration, and synaptic plasticity. These functions have the overall aim of building a functional brain. They can, however, cause cellular, biochemical, and structural alterations in the neonatal brain [30]. During brain development, the role played by neurotransmitters and their receptors is of primary importance. The distribution and molecular characteristics of Glu receptors change considerably during brain development, making the brain vulnerable to changes in Glu neurotransmission during growth. Indeed, alterations in the expression and regulation of Glu receptors are known to be implicated in some neuropathological conditions such as neurological and psychiatric disorders, including Alzheimer’s and Parkinson’s diseases, schizophrenia, mood disorders, depression, epilepsy, anxiety, stress, and ASD [31,32,33,34]. Studies in animal models of neurologic disease suggest an altered expression of Glu proteins [12,35]. Knowledge and research on the ontogenetic alterations in Glu receptor function, subunit expression, and binding properties in pathological conditions are still very incomplete. However, in vivo and in vitro studies have provided information about the differences in both regional densities and the time-course of changes in the expression levels of the different Glu receptors subunits (Figure 1) [36].

For example, the α-amino-3-hydroxy-5-methyl-4-isoxazolepropionic acid receptor (AMPAR) is constituted by tetramers of different subunits (GluR1-4) [37]. The expression pattern of the GluR2 subunit during the period of postnatal development, maturation, and aging in male mice was investigated: it was observed that the expression of the GluR2 subunit is gradually upregulated in the hippocampus from postnatal day 0 (P0) to adult age (20 weeks) and subsequently down-regulated in 70 week-old male mice [38]. GluR1 and GluR4 subunits are expressed at significant levels in the midbrain from embryonic day 15 (E15), they remain constant until delivery, and then decrease immediately after birth, while GluR2 and GluR3 levels increase after early development in the hippocampus [34]. N-methyl-D-aspartate receptors (NMDAR) are expressed during the developmental periods of intense synaptic formation. Similar to AMPAR, the expression of the various NMDAR subunits varies over the course of development and in different regions of the brain [39]. Studies in rodents have shown that the GluN1 subunit is expressed before E14 in areas of the brain related to cognitive function, such as the cortex, hippocampus, and thalamus. Three weeks after birth, GluN1 levels reach their highest levels and then decrease [40]. The GluN2 subunits are expressed at variable levels at specific stages of neonatal development in rats. In the hippocampus, low levels of GluN2A are detected at P0, while a peak is observed at P21 [41]. As the brain develops, the protein expression of the GluN2A subunit gradually increases. GluN2B is the predominant NMDA receptor subunit located at immature synapses [36]. During prenatal development, it is expressed in the cortex, thalamus, and spinal cord, and its concentrations increase at birth. Lower amounts are expressed in the colliculi, hippocampus, and hypothalamus [34]. After birth, GluN2B levels reach a peak of expression in the cortex and hypothalamus, two important brain structures involved in cognition. The GluN3A subunit is involved in developing dendritic spines and synaptogenesis [34]. This subunit starts to be expressed in the medulla, pons, and hypothalamus at E15 [42,43]; its levels reach a peak at P8 and then decrease at P20. Kainate-2-carboxy-3-carboxy-methyl-4-isopropenylpyrrolidine receptors (KAR) are expressed at low amounts from E19, during embryonic development, in the cortex, hippocampus, cerebellum, and striatum [34]. Their activation regulates the network and synaptic activity in the neonatal hippocampus [44,45]. KAR are widely expressed in the amygdala within the first postnatal week, a period that coincides with the process of synaptogenesis, suggesting its involvement in the process of synapse formation [45]. Rodent studies have demonstrated a low expression at birth of type 1, 2, and 4 Glu metabotropic receptors (mGlu1, mGlu2, mGlu4) that increase during neonatal development [43]. Conversely, at P0, the levels of type 3 and 5 Glu metabotropic receptors, mGlu3 and mGlu5, are very high and then decrease during the maturation period. In fact, both receptors are involved in synaptogenesis, and GRM5 is involved in the proliferation and survival of neural progenitor cells, as well as in the migration of cortical neurons [46]. Another important aspect of the brain development process is the maintenance of the excitation-inhibition (E/I) balance between Glu and Gamma-Aminobutyric Acid (GABA) [47]. Opposite to Glu, GABA is involved in inhibitory neurotransmission [48]. However, at the beginning of development, GABAergic neurons form excitatory synapses, which become inhibitory only later during the maturation process [49]. For normal brain development and functioning, it is essential that the E/I balance is maintained stably in neuron’s synapses and neural circuits [10,50]. Consequently, disturbances in the E/I balance have been implicated in neurodevelopmental disorders, including ASD [51,52].

## 3. Sex Differences in the Glutamatergic System

Accumulating evidence has revealed that gonadal steroids finely modulate neurotransmission mediated by amino acids, including Glu, in developing and adult brains [53]. Indeed, there appear to be functionally relevant baseline sex differences in neurotransmitter systems [11,54]. Clinical studies have highlighted increased Glu levels in frontal grey matter and basal ganglia (BG) of females compared to males, while parietal grey matter (PGM) Glu concentration is higher in men than in women [55]. Further studies have identified in more detail sex differences in Glu concentration in specific brain regions. Females were found to exhibit higher Glu levels than males in the sensorimotor cortex, anterior cingulate cortex (ACC), striatum (STR), and cerebellum [56,57]. In contrast, prefrontal cortex (PFC) Glu concentration is higher in males than in females [58]. Along with the Glu concentration changes observed in the CNS, gender differences were also found in plasma Glu concentrations. Clinical studies reported higher Glu levels in males than females, which appear inversely correlated to estrogen and progesterone levels [59]. Gender differences in Glu concentration appear to become more conspicuous with age. Males, but not females, display an age-dependent decline in BG and PGM Glu levels [55], while females show a more pronounced age-dependent decrease in ACC Glu levels [60]. In addition, blood Glu levels exhibit an age-related increase in plasma in females but not in males [61]. Similar to what is seen in humans, preclinical studies have revealed significant sex-related differences in the Glu system of rodents. Increased Glu levels have been found in the lateral hypothalamus and habenula of male rats [62]. Additionally, rodents show a sex bias in Glu receptors distribution. For example, female rats show an enhanced expression of mGlu2/3, mGlu5, and the GluN1 and Glu2B subunits of the NMDAR in the hippocampus [63,64,65], along with greater mGlu5 expression in the PFC [66]. In line with the reported sex-related differences in the rodent Glu system, in female rats, AMPAR-dependent synaptic events are altered: synaptic transmission is enhanced, whereas the long-term potentiation (LTP) magnitude is reduced, the latter alteration likely due to reduced phosphorylation of the GluR1 subunit [67].

Sex-specific factors have been hypothesized to increase male’s risk of ASD, or increase the protection of females from ASD, as more males than females are affected. The sex-related differences in the Glu system discussed above suggest the importance of elucidating the molecular mechanisms by which Glu dysfunction could differentially affect males and females.

## 4. Glutamate Signaling in ASD

Glu is the main excitatory neurotransmitter in the mammalian brain. The Glu concentration in the CNS has been estimated at around 10 mmol/L in the intracellular compartment [68], a much higher level than in the extracellular fluid (0.5–2 μmol/L) [69,70], in the cerebrospinal fluid (10 µmol/L) or plasma (150 µmol/L) [71]. As perturbations in the Glu system can have deleterious effects, brain Glu concentration is tightly regulated by a large number of mechanisms, such as the endothelial cells of the blood-brain barrier (BBB) and the Glu/glutamine (Glx) cycle between neurons and astrocytes, just to name a few. A large body of literature describes changes in both peripheral and brain levels of Glu and glutamine in ASD patients [72,73]. The first clinical reports, dating back to the 1990s, focused on plasma Glu levels. Using high-performance liquid chromatography (HPLC), Moreno et al. reported a significant increase in Glu and aspartate levels in children with ASD compared to controls [74,75]. The following study additionally reported reduced levels of plasma glutamine [74,75]. Notably, increased levels of plasma Glu and decreased levels of blood glutamine were also reported in siblings and parents of patients with ASD [18]. Since these first reports, many more described higher levels of plasma Glu and lower levels of plasma glutamine in children or adults with ASD compared to healthy controls [76,77,78,79,80,81,82,83,84,85,86]. Higher plasma levels of Glu have been associated with increased severity of ASD [82]. Similarly, it has been described as an elevated Glx ratio [87] in affected patients. It is worth noting that a few papers have shown a reduction or no change in blood or serum Glu concentration [8,73]. Altered Glu levels have been reported in plasma but also in brain regions of patients with ASD [8]. Although Glu does not enter the brain in appreciable quantities because of saturable and stereoselective facilitative transporters on the luminal membranes [70], blood Glu levels could, in principle, influence the brain concentration [73]. Despite Takado et al. demonstrating a positive correlation between plasma and brain levels only for glutamine but not for Glu [88], both these amino acids are metabolically engaged in a tightly balanced and highly dynamic cycle to maintain the proper concentrations of Glu and GABA [50,89]. In-vivo measurements of Glu levels in the human brain have been obtained by magnetic resonance spectroscopy (H-MRS). Because the molecular structures of Glu and glutamine are very similar, Glu spectral features are usually contaminated by contributions from glutamine; therefore, usually, the concentration of both amino acids is evaluated. Such work showed significantly higher concentrations of Glx in the amygdala-hippocampal region [90], primary sensory cortex [91], anterior cingulate cortex (ACC) [92,93,94], and auditory cortex [95], and a trend for lower Glu in the right medial temporal lobe [93], in people with ASD. Moreover, Siegel-Ramsay et al. reported an association of the increased Glx concentration with reduced functional connectivity between ACC and insular, limbic, and parietal cortical regions in patients with ASD [96]. However, some works reported opposite results, specifically a reduction of Glx or Glu concentration in the right ACC [97,98,99] basal ganglia [100] and cerebellum [98,101] in patients with ASD compared to healthy controls. Although with some discrepancies, overall, the data summarized above suggest an increase in Glu concentration in patients with ASD. In a healthy adult brain, both Glu and GABA maintain the excitatory/inhibitory (E/I) balance [102]. An enhanced Glu concentration causes prolonged activation of Glu receptors that may, in turn, produce a calcium overload in neurons. This phenomenon is known as excitotoxicity [103]. Several lines of evidence suggest that glutamatergic dysfunction, excitotoxicity, and neuroinflammation represent intertwined phenomena [104]. Indeed, cortical infusion of NMDA increased the expression of the pro-inflammatory cytokines tumor necrosis factor-alpha (TNF-α) and Interleukin-1beta (IL-1b) [105]. Accordingly, administration of an NMDA antagonist reduced both IL-1b and TNF-α levels [106]; on the other hand, an interleukin-1 receptor antagonist reduced excitotoxicity [107]. Glia and immune cells control pro-inflammatory activity driven by “on” and “off” signals originating from neurons. Glu release is an “on” signal that activates the release of the pro-inflammatory cytokines TNF-α and IL-1b [108]. While, on the one side, Glu can increase cytokine release, on the other side, cytokines seem able to reinforce Glu release. Indeed, the expression of astroglial high-affinity Glu transporters, contributing to Glu removal, is reduced during neuroinflammatory processes [109]. Furthermore, Glu release and accumulation are controlled by both IL-1b, by increasing Glu export via the cysteine/Glu exchanger [110] and TNF-α signaling [111]. Finally, recent evidence suggests that cytokines can induce ionotropic glutamatergic receptors mobilization. Indeed, TNF-α was shown to enhance excitatory synaptic strength through the exocytosis of AMPA receptors [112] and to decrease inhibitory synaptic strength by inducing the endocytosis of GABA(A) receptors [113], overall moving the E/I balance towards excitation. IL-1b was reported to boost NMDA receptor function by promoting GluN2A/B subunit phosphorylation [114] and inhibit GABA(A) receptor function [115], but other work showed that it down-regulates AMPA receptor surface expression [116].

## 5. The Genetics of the Glutamatergic System in ASD

The list of genes implicated in ASD susceptibility gets constantly richer, thanks to the large-scale genetic studies conducted on affected patients and their families. Because Glu receptors are highly enriched in brain areas associated with ASD, it is not surprising that mutations in Glu receptor genes and modifications in mRNA and protein expression of Glu receptors subunits have been found in patients. For the purpose of this review, we screened the Simons Foundation for Autism Research Initiative (SFARI) database (https://gene.sfari.org/, accessed on 31 January 2022), which includes hundreds of ASD candidate risk genes, to provide a comprehensive picture of the glutamatergic genetic risk factors for ASD (Table 1). The SFARI database is organized into four categories reflecting the overall strength of the available evidence supporting a gene’s relevance to ASD risk. The syndromic category includes mutations that are associated with a substantial degree of increased risk and consistently linked to additional characteristics not required for an ASD diagnosis. In this category, only the SLC1A2 gene, encoding for a glial high-affinity Glu transporter, is involved in the Glu neurotransmission system. Studies by Xu et al. have shown that mutations within the SLC1A2 gene occurred in ASD patients more frequently than in those without ASD [117]. Genes in category 1 are considered highly confident because they have been clearly implicated in ASD. Only two genes related to Glu neurotransmission are included in this category: GRIA2, encoding for the subunit type 2 of AMPAR [118,119,120], and GRIN2B, encoding for regulatory subunits type 2B of NMDAR [121,122,123,124]. Heterozygous de novo GRIA2 variants have been reported in patients diagnosed with intellectual disability and neurodevelopmental abnormalities, including ASD [125]. Further evidence has been provided by Ramanathan et al., who observed, in an autistic patient, a 19 megabase deletion in the chromosomal region containing the GRIA2 gene [126]. Sequencing studies of GRIN2B in ASD patients have identified a significant excess of rare missense mutations [127,128,129], severe de novo splice-site variants, and three additional de novo loss-of-function variants [130,131]. Further analysis for de novo mutations has confirmed GRIN2B as a high confidence candidate ASD gene [132,133,134]. Category 2 includes genes with two reported de novo likely-gene-disrupting mutations that have been implicated in ASD by a genome-wide association study. We have identified seven Glu genes in this category. GRIA1, encoding for the subunit type 1 of AMPAR, is a strong ASD candidate because a missense variant (p.Ala636Thr) has been identified in affected patients diagnosed with intellectual disability, developmental defects, and ASD [121,135,136]. Similarly, GRIN2A, encoding for regulatory subunits type 2A of NMDAR, has often been associated with neurodevelopmental phenotypes and ASD [121,123,124,135,136]. Interestingly, rare pathogenic deletions of the GRIN2A gene [137,138,139,140,141,142] and heterozygous de novo missense variants [143] have been implicated in childhood focal epilepsy. Moreover, next-generation sequencing of children with ASD has revealed variants in the GRIN2A gene with evidence that supports ASD pathogenicity [144,145]. Genetic studies have also found a genetic association between ASD and GRIK genes belonging to the kainate family of Glu receptors. In particular, GRIK2, encoding for kainate subunit type 2, and GRIK 5, encoding for kainate subunit type 5, have been classified as strong ASD candidates. A linkage disequilibrium has been revealed between ASD and GRIK2 in a mutation screening in a Chinese family [21,146]. Regarding GRIK5, three de novo missense mutations have been identified in children with ASD [147,148]. An association with ASD has also been found for the GRIP1 gene, encoding for a scaffold protein mediating trafficking and membrane organization of several transmembrane proteins. In particular, rare but high-incidence missense mutations have been reported in 480 cases compared to 480 controls [149]. Finally, ASD genome-wide copy number variation analysis reveals mutations in GRID1, the gene encoding for the Glu ionotropic receptor delta type subunit 1, in affected patients [150,151]. The last category comprises candidate genes with significant but unreplicated evidence of association with ASD or evidence lacking a rigorous comparison with healthy controls. GRM5 and GRM7 genes, encoding for Glu metabotropic receptors 5 and 7, respectively, are included in this category. However, there are some additional genes not included in the SFARI database. Indeed, genome-wide association studies provided evidence of a linkage between autistic disorder and variants in the chromosomal region 7q21–32, containing the Glu metabotropic receptor 8 gene (GRM8) [20]. Additionally, a microdeletion in the chromosomal region 7q31.33q32.1, containing 13 genes, including GRM8, has been found in a child with ASD and intellectual disability [152]. 

## 6. Glutamatergic Gene and Protein Expression in ASD

Post mortem studies examining the brains of individuals with ASD have pointed out the presence of alterations in Glu receptor expression. Significantly decreased AMPAR density and upregulated NMDAR subunit 1 protein levels have been observed in the cerebellum [22]. On the contrary, Blatt et al. failed to find differences in NMDAR expression in hippocampal tissue from 4 ASD patients [159]. Changes in metabotropic Glu receptors have also been described. Increased levels of mGlu5 receptor have been found in the cerebellar tissue of 11 affected patients [160]. Similarly, individuals with Fragile X syndrome (FXS), frequently co-diagnosed with ASD, showed increased mGlu5 protein expression in the prefrontal cortex compared with matched healthy controls [161]. A reduction in glutamic acid decarboxylase (GAD) has been reported in the parietal and cerebellar areas of affected patients compared to healthy controls [162], a deficiency that may account for the dysfunction in blood and brain Glu levels. GAD is a rate-limiting enzyme in the Glu/GABA cycle, converting Glu to GABA, and exists as two isoforms, GAD65 and GAD67 [163]. GAD67, whose gene is localized in a locus on chromosome 2 that showed susceptibility for ASD [164], is abundantly expressed in cerebellar Purkinje cells. Interestingly, a significant reduction of GAD67 mRNA levels in Purkinje cells of affected patients has been pointed out [165]. Notably, also the number and size of Purkinje cells were decreased in autistic patients [166]. In a further study, Yip et al. demonstrated a significant reduction of GAD65 mRNA in large-sized neurons of the dentate gyrus in children with ASD [167]. Increased mRNA levels of the excitatory amino acid transporter (EAAT) 1 and EAAT2 have also been described in the cerebellum of ASD patients [22]. The increased Glu concentration observed in plasma and spectroscopic studies are likely contributing to the enhanced EAAT expression [168]. Histological studies on brains of patients with ASD revealed small-sized neurons and increased neuronal packing in the limbic regions, including the hippocampus [169]. Aylward et al. reported a reduced volume of the amygdala and hippocampus in autistic patients with respect to the whole brain [170]. A subsequent study by Raymond et al. (1996) utilizing Golgi staining reported a reduced complexity in the dendritic branching of hippocampal pyramidal neurons, suggesting a curtailment in neuronal maturation [171].

## 7. The Role of Epigenetics in ASD

Epigenetics can provide insights into genetic and environmental disease risk factors and gene–environment interactions but also contribute to identifying disease-relevant genomic regions and biomarkers [172]. Epigenetic modifications appear to be strongly associated with ASD, suggesting that not only the genome but also the epigenome plays a role in ASD pathogenesis [173]. Indeed, several ASD-linked genes show epigenetic changes, such as DNA methylation or post-transcriptional histone modifications [174]. Increasing evidence suggests that early life environments may play a key role in ASD and other neurodevelopmental disorders. Epigenetics represent prime mediators of the environmental effects on genome and phenotype, and modifications caused, for example, by viral infections or the use of some drugs during pregnancy, can drive derangements of the gene regulatory processes during development [175,176,177]. There are four different mechanisms of epigenetic modifications that collectively play essential roles in gene transcription and expression: histone modifications, DNA methylation, RNA interference, and RNA modifications [178]. Epigenome-wide association studies (EWAS) on neurodevelopmental diseases focused primarily on the DNA methylation mechanism; conversely, less is known about histone modification changes [176,178,179,180]. Glutamate decarboxylase 1 (GAD1, aka GAD67), encoding an enzyme that catalyzes GABA production, is an example of epigenetic gene modifications associated with ASD [176,181]. Increased DNA methylation of the GAD1 gene promoter, accompanied by increased binding of the transcription repressor Methyl Cpg 2 (MeCP2), was described in the cerebellum of patients with ASD [182]. Accordingly, GAD1 expression was reduced in two rat models of ASD, the Poly(I:C)-induced maternal immune activation model [183] and rats prenatally exposed to the anticonvulsant valproate [184]. In addition, murine models with haploinsufficiency [185] or loss of GAD1 show deficits in social behavior and learning [186]. Interestingly, ASD is frequently associated with both an abnormal process of methylation and significantly differential DNA methylation patterns [176,187,188]. DNA methyltransferases (DNMTs) [176,188] polymorphisms seem involved in the pathogenesis of ASD [189]. The DNMT family includes three different enzymes, DNMT1, DNMT3a, and DNMT3b, playing a fundamental role in diverse cellular processes, such as neurodevelopment, synaptic plasticity, long-term memory learning, and training [187,190]. Accordingly, a reduced expression of DNMT1 or DNMT3a in excitatory neurons of the mouse forebrain causes alterations in synaptic plasticity, learning, and memory processes [191]. Excessive DNMT1 activity [192] or increased methylation of MeCP2 promoter lower the expression of MeCP2 [193] and consequently induce the GABA/Glu imbalance that might be involved in ASD pathophysiology [194,195]. Several studies have shown that epigenetic changes can alter the expression of pre- and postsynaptic proteins involved in ASD, such as Neurexins [196], Neuroligins (NLGNs; [197], PSD-95 [190], and SHANK3 [187]. There is experimental evidence describing an association between a differential methylation state of the promoter region of these genes and their downregulation, and genetic variations of these proteins have been implicated in ASD as responsible for the appearance of a variety of symptoms such as cognitive and social deficits and anxiety [172]. Despite conflicting evidence about the role in ASD pathogenesis of epigenetics, it may mediate or modify the genetic or environmental disease risk or represent the biological mechanism for gene-environment interactions [198]. On the other hand, it could be possible to identify epigenetic modifications as biomarkers of exposure to environmental risk factors [199].

## 8. Role of Glutamatergic Transmission in ASD Pathophysiology: Evidence from Animal Models

**Figure 2:**
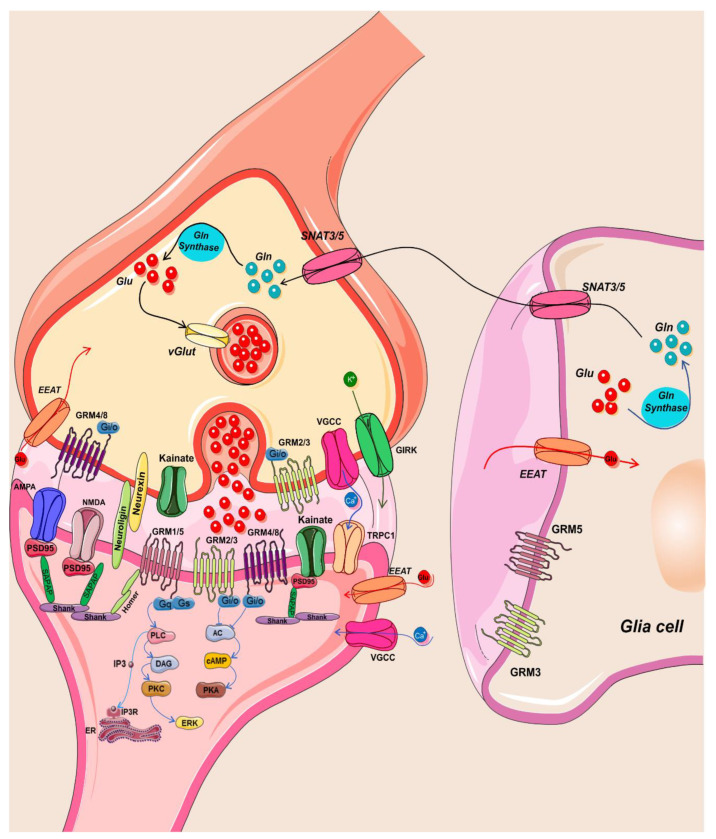
Schematic drawing of the glutamatergic synaptic pathway altered in ASD. Once released, glutamate can bind to and activate AMPA, Kainate, and NMDA ionotropic receptors or G-protein coupled metabotropic receptors on postsynaptic or presynaptic neurons as well as glial cells. Glutamate, taken up into glial cells by EAAT1/2 glutamate transporters, is converted into glutamine, which can be transported into glutamatergic neurons by SNAT3/5 transporters.

Alterations in Glu expression levels, reduced excitatory transmission, dysfunction of NMDAR-mediated synaptic plasticity, and mGluR-mediated signal transduction play a role in the cognitive and behavioral deficits of ASD [9]. Both ASD patients and animal models show an altered expression of synaptic proteins as well as dysfunction of excitatory or inhibitory neurons [52,200,201]. These observations gave rise to the theory of the E/I imbalance in the pathophysiology of ASD ([52,200,201]. Indeed, several ASD models, such as the Fmr1 [202] and the Cntnap2 mice [203], as well as the VPA-induced model, [204,205]) show a reduction in GABAergic interneurons numbers [206]. An in vivo optogenetic study provided for the first time a demonstration of the relationship between alterations in the E/I balance in the prefrontal cortex of WT mice and ASD-associated behaviors [207]. In addition, a highly significant correlation between a decreased Glu/GABA ratio in the prefrontal cortex and mice sociability has been recently demonstrated in the Cntnap2^−/−^ mouse model [208]. Such alterations might be caused by reduced inhibitory postsynaptic currents (IPSC) and/or increased excitatory postsynaptic currents (EPSC), as observed in the Cntnap2^−/−^ [209,210] and the VPA [211] model. Preclinical studies have shown a clear role of Glu in ASD-like behaviors. Indeed, glutamatergic neurotransmission dysfunctions have been demonstrated in various animal models (Table 2) [212,213] In particular, the regulatory proteins of the glutamatergic system (Figure 2), including Glu receptors (such as GluK2, mGlu8, NMDAR, GluR1, and GluR2 Glu receptors) and Glu transporters (such as glial Glu transporters, GLT1, GLAST, SLC1A1, and EAAT-1,2) are highly implicated in the pathophysiology of the disorder [10].

### 8.1. AMPA Receptor

Deficits at the level of AMPAR-mediated currents have been identified in diverse mouse models of ASD [201,232,233]. Shank2 (Shank2^−/−^) and Shank3 (Shank3αβ^−/−^) knock-out (KO) mice exhibit reduced levels of several cell surface Glu receptors in the striatum and thalamus: additionally, Shank3αβ^−/−^ KO mouse models showed alterations in AMPAR expression [234]. Similarly, neuronal cultures of the embryonic brain stem from NLGN1-2-3 KO mice showed deficits in AMPAR-mediated glutamatergic transmission, namely alterations in the frequency of spontaneous and miniature excitatory synaptic currents (sEPSCs and mEPSCs) [227]. The R704C-NLGN3 mice show reduced AMPAR currents in the hippocampus [229]. It has been shown that hippocampal CA1 pyramidal neurons in the Fmr1-KO mouse model exhibit a higher AMPAR/NMDAR ratio early in development, returning to normal levels at P13. This early alteration is accompanied by increased expression of the GluR2 subunit in synaptic AMPARs leading to altered Ca2+ permeability [217,218]. Additionally, in FMR1 KO mice, a high expression of the AMPAR subunits GluR1-4 was observed in the hippocampal CA1 pyramidal neurons and Purkinje cells of the cerebellum [214,215], where anatomical alterations have also been observed [235].

Alterations in Glu receptors expression and transmission have been demonstrated both in the Cntnap2 KO model and in mice prenatally exposed to valproic acid (VPA); however, while an enhanced GluR1 and GluN2B expression was observed in VPA mice, a decrease in the subunits GluR1, GluR2, GluN2A, and GluN2B was reported to occur in Cntnap2 KO mice [52]. In line with such evidence, AMPAR modulation improved social deficits in Cntnap2 KO and VPA animal models [52]. Accordingly, two ampachins, CX1837 and CX1739, are drugs acting at AMPARs tested on BTBR mice, an inbred model line with ASD-like behaviors, which showed a selective improvement in social behaviors with no effect on self-grooming [231].

### 8.2. NMDA Receptor

Several animal models of ASD show impairments of NMDAR signaling [216,221,236,237], including SHANK3 [225], NLGN1-KO [238], FMR1 KO [216], and transgenic MeCP2 mice [239]. Increases in NMDAR expression were found in a prenatal VPA rat model [221], while lower levels were found in the FMR1 KO mouse [216] and the MeCP2^−/−^ mouse model [240]. In the NLGN1 KO model, the NMDAR co-agonist d-cycloserine reduced anxiety and repetitive behaviors, suggesting that the increased NMDAR activity plays a role in some ASD-associated symptoms [228]. In addition, the increased expression of NMDAR in rats exposed to VPA is accompanied by enhanced long-term potentiation, reduced sensorimotor gating, repetitive/stereotyped movements, and abnormal social behaviors [221,222]. The NMDA/AMPA ratio was increased in the pyramidal neurons of the hippocampal CA1 region in NLGN3-R451C KI but not in NLGN3 KO mice. The R451C mutation caused an increase in the expression of the excitatory postsynaptic scaffolding proteins PSD95 and SAP-102, and of the GluN2B subunit of the NMDAR, without affecting AMPAR [230]. Emerging evidence suggests that disrupted NMDAR-mediated signaling may contribute to the pathogenesis of idiopathic ASD [9,127] and related syndromes, including Rett and Fragile X [240]. In support of this view, GluN1 hypomorphic mice, in which GluN1 expression is reduced to 5–10% of control levels, show deficits in social interaction [241]. GluN2A, which encodes one of the subunits of the NMDA receptor, has also been studied in relation to the ASD spectrum as it is located on chromosome 16p, a region identified as a possible ASD susceptibility locus [10,123].

### 8.3. KA Receptor

The physiological role of KAR is not characterized as for other Glu receptors, but it is clear that it is involved in the maturation of neural circuits during development [242]. Chromosome 6q21 is a region that contains the gene coding for the GluK2 subunit of KAR: this subunit is an excellent positional and functional candidate for ASD susceptibility [21]. GluK2 is known to play a role in the appropriate maturation of synaptic circuits involved in learning and memory [243]. GRIK2 KO (GRIK2^−/−^) mice have been shown to exhibit both functionally and morphologically delayed maturation of the hippocampal mossy fiber at CA3 (mf-CA3) pyramidal cell synapses; this mutation results in a transient reduction in the amplitude of AMPA-EPSCs at a critical time in postnatal development, affecting the proper formation of memory-related neural circuits [244]. In addition to this, it was observed that GRIK2^−/−^ mice show ASD-like behaviors: deficits in social interaction, reduced locomotor activity, and impaired spatial reversal learning [245]. A mouse model overexpressing the KAR subunit GRIK4 showed a marked decrease in social interactions, accompanied by enhanced anxiety and depressive states. In addition, this model showed synaptic transmission alterations at hippocampal mossy fibers, as well as in the basolateral and central nuclei of the amygdala [246]. In vivo results show anxiety and social interaction deficits, as well as in vitro an alteration in synaptic transmission. These data support the hypothesis that increased expression of the GluK4 subunit of KAR may contribute to ASD-associated behaviors [247].

### 8.4. MGlu Receptor

Genetic studies indicate a complex and heterogeneous etiology of ASD, although many ASD-linked genes appear to converge on the pathway of synaptic transmission and synapse formation [248,249,250]. One of the common aspects of many animal models of ASD involves altered forms of synaptic plasticity mediated by glutamate metabotropic (mGlu) receptors: this was seen in animal models of Fragile X (FMRP) [219], SHANK3 [226], tuberous sclerosis (TSC1/TSC2) [251], PTEN [252], 16p11.2 16 microdeletion [253] and Rett syndrome (MECP2) [254]. Both mGlu1 and mGlu5 belong to group I metabotropic receptors. In the context of ASD, particular attention was devoted to mGlu5, in light of its role as a regulator of both natal and postnatal neurogenesis and synaptogenesis [255,256], as well as in motor and social behaviors that are specifically affected in ASD disorders [236], suggesting that dysregulation of mGlu5 signaling could be involved in the pathogenesis of neurodevelopmental disorders. At the behavioral level, mGlu5 is involved in locomotor reactivity to novel environments, sensorimotor gating, anxiety, and cognition [257]. MGlu5 KO mice show alterations in the behavioral domains affected in ASD, such as burial behavior, social interaction, locomotor activity, and anxiety [258]. Accordingly, alterations in mGlu5 have been associated to diverse neurological and neuropsychiatric disorders, including FXS [220,259], attention-deficit/hyperactivity ADHD [260], ASD [155], schizophrenia [261,262] and epilepsy [32]. An overactive mGlu5 signaling is thought to be responsible for multiple behavioral features of FXS [219,220]. Interestingly, the synaptic, biochemical, and cognitive defects observed in the TSC2^+/−^ and FMR1^−/y^ mouse models were corrected by treatments that modulate mGlu5 in opposite directions, suggesting that normal synaptic plasticity and cognition occur within an optimal range of mGlu5 mediated effects, and deviations in either direction can lead to shared behavioral impairments [263]. The potential role of mGlu1 has been characterized limited to cognitive aspects in mouse models of ASD. The mGlu1 antagonist, JNJ16259685, restores social deficits in Eif4ebp2 knockout mice but not in Shank2 KO rats [224,264], while CFMTI, another selective mGlu1 antagonist, ameliorates the social interaction deficits induced by the NMDAR antagonist MK-801 in rats [265]. With regard to mGlu antagonists, numerous preclinical studies have shown their key role in treating ASD. FXS pathophysiology is characterized by excessive mGlu signaling that contributes to the behavioral, electrophysiological, and molecular dysfunction associated with the disorder [266]. Preclinical studies in FMR1 KO animal models showed a restoration of aberrant AMPAR expression, behavioral deficits, electrophysiological alterations, protein expression dysregulation, and altered dendritic spine morphology [267] through the use of 2-methyl-6-(phenylethynyl) pyridine (MPEP), a selective mGlu5 antagonist [234].

## 9. GABA/Glutamate Balance in ASD

As the correct balance between glutamatergic and GABAergic neurotransmission is essential for the normal information processing, stability, and organization of the neuronal networks [207,268], an imbalance between excitation and inhibition, consequence of dysfunctions in terms of potentiation of glutamatergic signaling, and/or weakened GABAergic signaling might be implicated in ASD pathophysiology [200,269,270,271]. GABAergic transmission plays a pivotal role in cellular functions, most importantly during brain development. Indeed, in the embryonic and early postnatal period, GABA exerts an excitatory action on immature neurons and, in synergy with Glu, drives glutamatergic synapse development, cell migration, and differentiation [272,273]. As the developmental depolarizing-to-hyperpolarizing switch in GABA transmission is critical for the correct establishment of the E/I balance [274], alterations in GABAergic signaling can impair normal cellular processing and cause deficits such as those observed in ASD [275]. To date, increasing evidence of dysfunction in the GABAergic neurotransmission system has been reported in patients and animal models of ASD [276,277]. In vivo 1H-MRS measurements have revealed a significant increase in plasma levels of GABA in children with ASD compared to controls [81,85,278]. Conversely, analyses of brain areas reported a reduced GABA/Glu ratio in the frontal lobes of autistic children [279], in the occipital brain areas of adolescent patients with high-functioning ASD [280], and the PFC of adult patients in response to riluzole [281]. Differing results showed either increased GABA levels in the visual cortex or reduced GABA levels in the sensorimotor cortex and reduced GABA/Creatine levels in ACC in children with ASD compared to controls [282,283,284]. Alterations in GABAergic signaling have been demonstrated by brain postmortem studies: namely, an increased expression of GAD67 and reduced expression of GAD 65 in cerebellar cells, an increased density of GABAergic interneurons in the hippocampus, and a downregulation of several GABAergic receptor subunits [167,285,286,287,288]. Furthermore, genetics added further evidence of the involvement of GABAergic dysfunction in ASD. Indeed, rare mutations, including de novo and intrinsic variants, have been found in the chromosome 15q11–q13 region, a site containing coding regions of specific subunits of GABA receptors, including GABRB3, GABRA5, and GABRG3 [289,290,291,292]. Finally, direct evidence of GABAergic dysfunction has been obtained from molecular and cellular studies in several genetic and pharmacological animal models of ASD [293,294,295,296,297,298,299,300,301,302,303,304,305,306]. Overall, the dysfunctions shown in both glutamatergic and GABAergic neurotransmission suggest a pathological switch in the E/I balance underlying, at least partly, the failure of compensatory mechanisms, such as adaptation of synaptic efficacy, plasticity, membrane excitability, and/or synapse numbers, physiologically implemented to prevent over-excitation [307]. Accordingly, in a recent article, Bruining et al. demonstrated an increased functional E/I ratio (fE/I) and long-range temporal correlations (LRTC) of the network activity in children with ASD by means of a computational model of neuronal network oscillations, also providing evidence about a correlation of these findings with the heterogeneity of ASD phenotype [308]. Similarly, Spiegel et al. provided indirect evidence of altered E/I balance in the visual cortex of patients with ASD using the experimental binocular rivalry test paradigm; interestingly, the slower rivalry dynamics in the ASD brain were associated with higher autistic traits [309]. Moreover, in a recent study, a positive correlation between the severity of the autistic phenotype and the magnitude of the E/I ratio was demonstrated using transcranial magnetic stimulation to monitor cortico-spinal excitability and intracortical inhibition [310]. Sex-specific mechanisms responsible for the high male/female ratio of ASD may differentially affect the E/I balance and, consequently, the ASD phenotype, as was recently demonstrated using a combination of in-silico modeling and in-vivo chemogenetic manipulations in mice [311].

## 10. Alterations of Glutamate Metabolism in the Gut

Increasing evidence suggests that Glu, in addition to being the main excitatory neurotransmitter in the brain, is an important neurotransmitter in the ENS and GI tract [312]. In the gut, Glu, which is derived from food and, in minor part, from microbial activity, is strongly involved in several functions, including taste perception and digestion process. Of the total amount of dietary Glu, 75–95% is rapidly removed to maintain a low concentration in the systemic circulation and prevent dangerous accumulation in other parts of the body [16]. In healthy conditions, dietary Glu does not pass the BBB. However, in pathological situations associated with diet or microbiota alterations, the BBB permeability may change, and Glu accumulates in the brain [313,314]. Enteric Glu strongly contributes to the bidirectional communication between gut and brain, via the activation of receptors distributed on vagal, splanchnic, and pelvic afferents, and vice versa, through the receptors on efferent pathways comprising the dorsal motor nucleus of the vagus (DMV) [315]. Interestingly, the administration of probiotics in mice can stimulate microbiota to modulate metabolic pathways and increase Glu brain levels [316,317]. In addition, it has recently been demonstrated that dysregulation in protein phosphorylation found in hippocampus tissue of mice with dysbiotic gut microbiota is consistently associated with glutamatergic neurotransmitter system disturbances [318]. Indeed, ultrastructural and immunohistochemical evidence demonstrates that ENS is provided with the whole glutamatergic machinery, including vesicular glutamatergic transporters [319,320] and all the known ionotropic (iGlu) and mGlu receptors [17,321]. Both iGlu and mGlu receptors have been found in the esophagus, stomach, small and large intestine, where they may participate in specific functions, including muscle activity and local blood flow [17,315,322]. Interestingly, numerous morphological and molecular similarities exist between enteric glia and astrocytes, particularly in glutamine synthase and iGlu receptors expression [323,324,325], suggesting that also in the ENS, glial cells may contribute to preventing extracellular Glu concentrations from rising to neurotoxic levels [326]. The gut microbiome composition and relative alterations are of particular interest in ASD research in light of numerous reports regarding dysbiosis and GI problems, such as diarrhea, constipation, abdominal pain, and gastric reflux, in children and adults with ASD. Recently, the characterization of the ASD gut microbiome highlighted dysregulations of the enteric glutamatergic neurotransmitter machinery that may have a role in the development of symptoms. A study on 43 ASD children found reduced gut cortisol levels associated with changes in Glu metabolism, with a significant reduction in 2-keto-glutaramic acid, and changes in the microbiota, with particularly low levels of *Bacteroides vulgatus* and high levels of both *Eggerthella lenta* and *Clostridium botulinum*, in fecal samples [327]. In line with this, a ketogenic diet, rich in fats and low in carbohydrates, was shown to reduce seizure susceptibility in two different mouse models of ASD through its impact on the gut microbiota composition, causing an increase in *Parabacteroides* and, in parallel, in brain Glu levels [328].

## 11. Pharmacotherapeutics Targeting the Glutamatergic System

Even today, the development of new therapeutic strategies is hampered by the extremely heterogeneous physiopathology of ASD and the scarcity of clear diagnostic markers [329]. In the United States, pharmacological interventions approved for ASD are limited to Risperidone and Aripiprazole, both of which are indicated by the Food and Drug Administration (FDA) for the treatment of irritability associated with ASD [330,331], but not for the core symptoms identified by the DSM-5 as essential for ASD diagnosis [332]. Therefore, it is crucial to find new pharmacological targets for effective pathogenesis-based treatments aimed at reducing symptoms, improving functioning, and potentially impacting long-term outcomes. Several ongoing clinical trials (https://clinicaltrials.gov, accessed on 31 January 2022) target a number of neurobiological pathways, GABAergic and Glutamatergic transmission, neuroinflammation, neuropeptides, and the endocannabinoid system. So far, Glu receptor antagonists have shown limited clinical efficacy in treating ASD. Drugs tested in these trials include memantine, an NMDA receptor antagonist, and mGlu5 antagonists, such as basimglurant, fenobam, and mavoglurant [8]. The phenotypic rescue demonstrated in FMR1 KO mice modulating mGlu5 activity has driven extensive clinical trial work since 2008. By means of a PubMed search, we identified 22 clinical studies, of which 19 (86%) are registered on www.ClinicalTrials.gov (accessed on 31 January 2022). Most of these studies (14/22, 64%) targeted the main excitatory/inhibitory imbalance primarily through mGlu5 antagonists or GABA agonists. Trials of three different mGlu5 antagonists—phenobam, mavoglurant (AFQ056), and basimglurant (RO4917523)—in FXS were completed. Although the alteration of E/I balance seems to be strongly associated with ASD, it is still not entirely clear how it can be modulated [333,334]. Functional magnetic resonance imaging (fMRI) was used to measure the functional connectivity of the prefrontal cortex following pharmacological treatment with the drug riluzole [281]. This drug has been used because it has a wide range of actions on both GABA and Glu targets [335,336] and consequently appears to be able to modulate Glu-GABA flux in most individuals. Riluzole shows a good pharmacokinetic profile and a very low risk of side effects; in addition, a single oral dose of 50 mg (as used in the study) reaches a peak plasma concentration 1 h after administration [337,338]: riluzole blocks the presynaptic release of Glu, facilitates GABA receptor activity and appears to be involved in the altered Glx balance in bipolar disorder [339]. Treatment with riluzole increased the inhibitory index of the prefrontal cortex in patients with ASD compared to controls and rescued the reduced prefrontal functional connectivity; notably, in controls, the decrease in GABA fraction was correlated with an increase in glutamine, whereas in ASD patients, riluzole increased the inhibitory index without altering glutamine [337,339]. In summary, riluzole shifts the balance towards GABA in ASD patients and towards Glutamine in controls. Riluzole acts by inhibiting voltage-dependent sodium channels, reducing neurotransmitter release, and favoring astrocytic uptake of extracellular Glu [340]. With regard to the pharmacological effects of riluzole, it was seen that long-term treatment (10–12 weeks) had a significant effect on irritability in young children with ASD [341], whereas long-term monotherapy (excluded medications, riluzole 50–200 mg/day for 8 weeks) additionally reduced the hyperactivity [342]. In contrast, in a group of children and young adults (aged 12–25 years), no pharmacological effect of riluzole was found [343]. There are many studies using memantine, a non-selective antagonist of NMDARs. Joshi et al. used memantine in a 12-week randomized trial to treat social deficits in ASD adolescents [344]. Neuroimaging, functional magnetic resonance imaging, and resonance spectroscopy analyses were conducted pre- and post-treatment to assess functional neuronal deficits in adolescents with ASD and healthy volunteers. A significant improvement in non-verbal communication was observed in the Diagnostic Analysis of Nonverbal Accuracy Scale test and the executive function for self-report. Memantine was well-tolerated without causing serious side effects [344]. Very often, high Glu levels correspond to hyperexcitability in the brain, which can be caused by an elevated level of Glu in the synapses and/or overexpression of its receptors. In this double-blind, placebo-controlled study, 28 children with a primary diagnosis of ASD were given lamotrigine, a drug that modulates Glu release. The children who received the treatment showed improvement in the language aspect of communication and socialization [345]. NMDA receptor antagonists appear to be effective in hyperexcitability and aggressive behavior [346]. Memantine has been hypothesized to play a role in potentially modulating learning, blocking the excessive effects of Glu, which may include neuroinflammatory activity, and influencing neuroglial activity in ASD [347]. Although the results of clinical trials of pharmacological treatments for ASD so far show promising results, outcomes remain highly variable in the treatment of core symptoms, and there is still no solid demonstration of efficacy involving one or more pathways or targets for one treatment over another. When designing a drug development plan for the treatment of core symptoms of ASD, it is critical to take into account that ASD is a neurodevelopmental disorder that spans the entire human lifespan and that efficacy and response to specific treatments may differ over this timeframe [345,348,349,350]. Although there are still doubts about the hierarchy of evidence supporting the safety and efficacy of such use, it is clear that the unmet clinical needs of individuals with ASD change over the course of a lifetime, suggesting that the relevance of specific classes of new (experimental) or old drugs (and the molecular targets through which they act) may vary over the course of a lifetime [345,348,349,350]. It cannot be assumed that the biology involved in new and different targets will be consistent at all stages of development. Therefore, it is reasonable that initial approval for an ASD treatment should include children and young adults. Similarities can be drawn with attention-deficit/hyperactivity disorder, where the FDA requires initial applications for new drugs to include children up to 6 years of age. Separate clinical trials in children and adults are likely to be required for drug development to ensure efficacy and safety in both groups. After initial approval, younger and older individuals with ASD could be studied as part of the post-approval effort or through other mechanisms [348,349,350].

## 12. Conclusions

ASD is a multifactorial neurodevelopmental disease caused by multiple etiology involving genetic, epigenetic, and environmental risk factors. Nevertheless, the congruence of ASD behavioral symptomatology and developmental stage suggests a common neurobiological-based etiology. Here, we examined the current literature supporting the possible involvement of glutamatergic dysfunctions in ASD pathophysiology as glutamate is directly involved in brain development and function as well as the bidirectional communication axis between gut microbiota and the Central Nervous System. The hypothesis that glutamate signaling is altered in ASD is supported by post-mortem research, clinical reports, and animal studies. In addition, the large-scale genetic studies conducted on affected patients and their families provide strong support for the idea that glutamate plays a major role in ASD pathophysiology. In the last decade, the prevalence of ASD has dramatically risen, although it remains unclear if this reflects an effective increase in occurrence or if better knowledge and more awareness of the autistic status have facilitated diagnosis. Indeed, it could be a concurrence of factors: the advancement of genetic research, the capacity for early identification of autistic signs, the development of new guidelines, and diagnostic criteria. In addition, recent years have brought the understanding that in addition to genetics, other factors play a role in ASD pathogenesis, such as epigenetic modifications, prenatal exposure to drugs or infections, immune dysfunctions, and alterations in the gut microbiome. Admittedly, there are still many aspects to be clarified in ASD. A first challenge remains to elucidate the enormous phenotypic heterogeneity, including adaptive function, cognitive and language abilities, and neurological comorbidities, and characterizes different disorders within the spectrum. Secondly, we still know very little about how the autistic condition evolves in adulthood or whether or not the main symptoms persist or change [351]. The diagnosis in adults is complicated by the lack of relevant information and the camouflage behaviors that adults, more than children or adolescents, can implement [352]. Also of concern is the limited availability of targeted therapies and/or the limited success in developing new medications. In this sense, identifying new biomarkers could greatly benefit the development of targeted and precision treatments. Clarifying how glutamatergic dysfunction contributes to the symptomatology of ASD might help to identify new pharmacological targets. To date, there are no approved glutamatergic drugs for ASD, although several clinical trials are in an early stage aimed at evaluating glutamatergic pharmaceuticals already approved for other conditions in patients with ASD. The pharmacological modulation of group I mGlu receptors may represent a potential therapeutic strategy for the treatment of ASD [353], and, indeed, some antagonists of mGlu1 and mGlu5 receptors were shown to rescue some phenotypes in ASD mouse models [264,354]. Nevertheless, thus far, figuring out how Glu biology is affected in ASD has proved to be extremely challenging. On one hand, the comorbidity of ASD with other Glu-related diseases, such as epilepsy, can provide excellent insights, but, on the other hand, the ASD heterogeneity still represents an important limitation in understanding how Glu changes can impact ASD pathophysiology. Moreover, glutamatergic dysfunction may significantly differ across the various disorders that characterize the Spectrum and, further complicating matters, glutamatergic transmission is strongly influenced by gonadal hormones, and basal Glu brain concentration differs between males and females. Finally, shedding light on how glutamatergic dysfunction changes and evolves from the earliest to the late stages of disease could provide information on the efficacy of drug treatment. In this context, clinical research, along with preclinical research on genetic and neurodevelopmental models, could prove extremely useful.

## Figures and Tables

**Table 1:** Candidate genes implicated in ASD.

Gene	Protein	SFARI Gene Score	Evidence	References
SLC1A2	excitatory amino acid transporter 2 (EAAT2)	Syndromic (Score 3)	-Rare de novo deletion, Genetic Association	[153]
GRIA2	Glutamate ionotropic receptor AMPA type subunit 2	High confidence (Score 1)	-Heterozygous de novo variants;-19 megabase deletion in the chromosomal region containing the gene	[119,120,125,126,154]
GRIN2B	Glutamate Ionotropic Receptor NMDA Type Subunit 2B	High confidence (Score 1)	-Rare missense mutations; -de novo splice-site variants -de novo loss-of-function variants	[127,128,129,130,131,132,133,134]
GRIA1	Glutamate Ionotropic Receptor AMPA Type Subunit 1	Strong candidate (Score 2)	-Missense variant (p.Ala636Thr)	[121,135,136]
GRIN2A	Glutamate Ionotropic Receptor NMDA Type Subunit 2A	Strong candidate (Score 2)	-Rare pathogenic deletions -Heterozygous de novo missense variant	[123,137,138,139,140,141,142,143,144,145]
GRIK2	Glutamate ionotropic receptor kainate type subunit 2	Strong candidate (Score 2)	-Rare Single Gene Mutation, Genetic Association	[21,146]
GRIK5	Glutamate ionotropic receptor kainate type subunit 5	Strong candidate (Score 2)	-Rare single gene mutations	[147,148]
GRIP1	Glutamate Receptor-Interacting Protein 1	Strong candidate (Score 2)	-Missense mutations	[149]
GRID1	Glutamate Ionotropic Receptor Delta Type Subunit 1	Strong candidate (Score 2)	-Rare Single Gene Mutation, Genetic Association	[150,151]
GRM5	Glutamate metabotropic receptor 5	Suggestive evidence (Score 3)	-De novo in-frame deletion variant, Genetic Association; -Twelve rare variants	[155,156]
GRM7	Glutamate metabotropic receptor 7	Suggestive evidence (Score 3)	gene variations and susceptibility to ASD; Rare de novo deletion	[157,158]
GRM8	Glutamate metabotropic receptor 8	/	-Variants in the chromosomal region 7q21–32 -Microdeletion in the chromosomal region 7q31.33q32.1	[20,152]

**Table 2:** Glutamatergic dysfunctions reported in pharmacological and genetic animal models of ASD.

Mouse Model	Alterations in Glutamatergic Signaling	Refs.
FMR1 KO (Fragile X)	High expression of the AMPAR subunit GluR1-4 in the hippocampal CA1 pyramidal neurons and Purkinje Cells of the cerebellum.	[214,215]
	Lower levels of NMDAR expression.	[216]
	Early transient increase in AMPAR/NMDAR ratio and increased expression of the GluA2 subunit in synaptic AMPARs.	[217,218]
	The mGlu5 antagonist MPEP improved NMDA-mediated deficits in LTP.	[8]
	Altered forms of synaptic plasticity mediated by overactivation of mGlu5.	[219,220]
VPA model	Alterations in E/I balance.	[207]
	Alterations in Glu receptor expression and synaptic transmission.	[52]
	Increased NMDA receptor expression and long-term potentiation.	[221,222]
SHANK2 KO	Reduced levels of several cell surface Glu receptors in striatum and thalamus.	[223]
	mGlu1 antagonist ineffective in rescuing the social deficits in SHANK2 KO rats	[224]
SHANK3 KO	Reduced levels of several cell surface Glu receptors in striatum and thalamus. Alterations in AMPAR expression.	[223]
	NMDAR dysfunction	[225]
	Altered forms of synaptic plasticity mediated by mGlu	[226]
NLGN1-2-3 KO	Alterations in AMPAR expression in brainstem neuronal cultures. Alterations in the frequency of spontaneous and miniature excitatory postsynaptic currents. No effect on evoked postsynaptic currents.	[227]
NLGN1 KO	The NMDA co-agonist d-cycloserine reduced anxiety and repetitive behavior	[228]
NLGN3-R704C	Altered AMPAR-mediated currents.	[229]
NLGN3-R451C	Increased NMDA/AMPA ratio in pyramidal neurons of the CA1 region of the hippocampus.	[230]
	Non-significant increase in the frequency of mEPSCs in the hippocampus.	
	Increased expression of the excitatory postsynaptic scaffolding proteins PSD95 and SAP-102, and the GluN2B subunit of the NMDA receptor	
BTBR	Decreased plasticity and excitatory postsynaptic potentials.	[231]
